# Supplementary material for: PTX-3 Secreted by Intra-Articular-Injected SMUP-Cells Reduces Pain in an Osteoarthritis Rat Model
Source: Cells. 2021 Sep 14;10(9):2420. doi: 10.3390/cells10092420 (PMC8466059; doi:10.3390/cells10092420)
Supplement: Supplementary file 1 [file cells-10-02420-s001.zip › cells-1353521-supplementary.pdf]

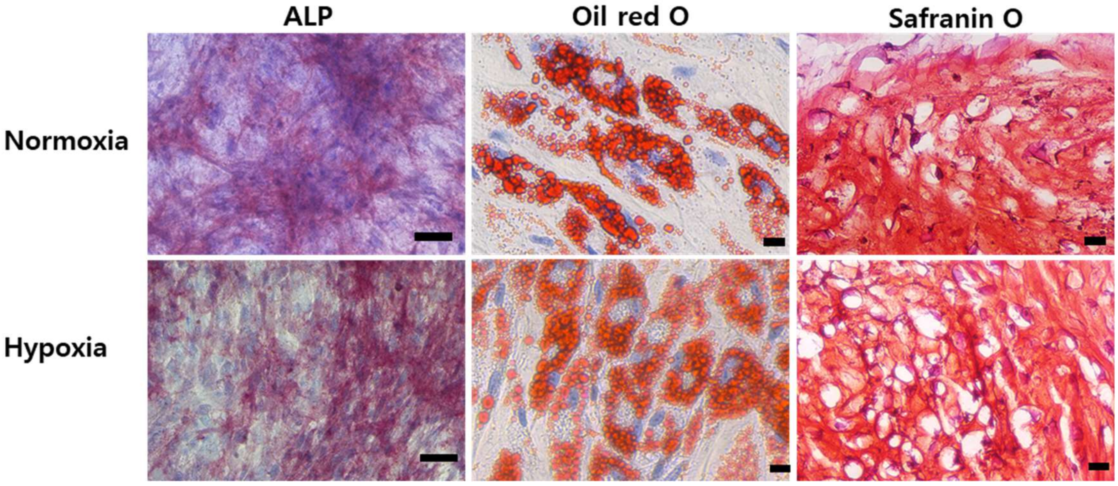

Figure S1. Multi-lineage differentiation. We prepared different condition of UCB-MSCs (normoxia vs. 3% hypoxia). After incubation in specialized induction media, cells showed multi-lineage differentiation based on staining for positive markers. Osteogenic cells were analyzed according to ALP level, adipogenic cells showed promoted lipid vacuoles within the cytoplasm via Oil Red O staining, and chondrogenic cells accumulated sulfated proteoglycans stained with Safranin O. Nuclei were counterstained with hematoxylin. Scale bar: 100 µm.

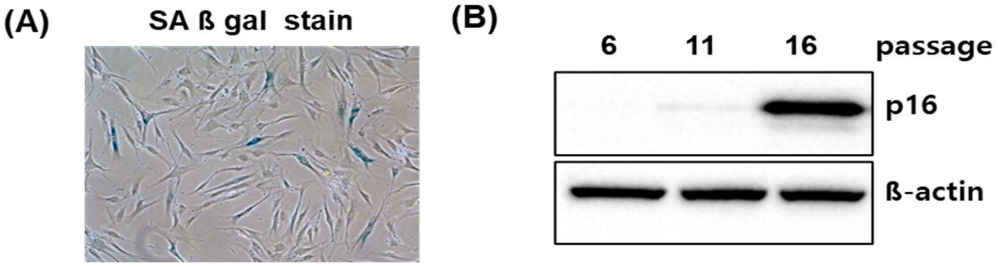

Figure S2. **Senescence phenotype of SMUP-Cell following expansion.** (A) The cells were stained to analyze SA ß gal expression at passage 16 using microscope (blue). (B) Senescence protein, p16 was measured by immunoblotting. The level of p16 expression increased during passage 6 to passage 16. Expression levels were normalized to ß-actin.

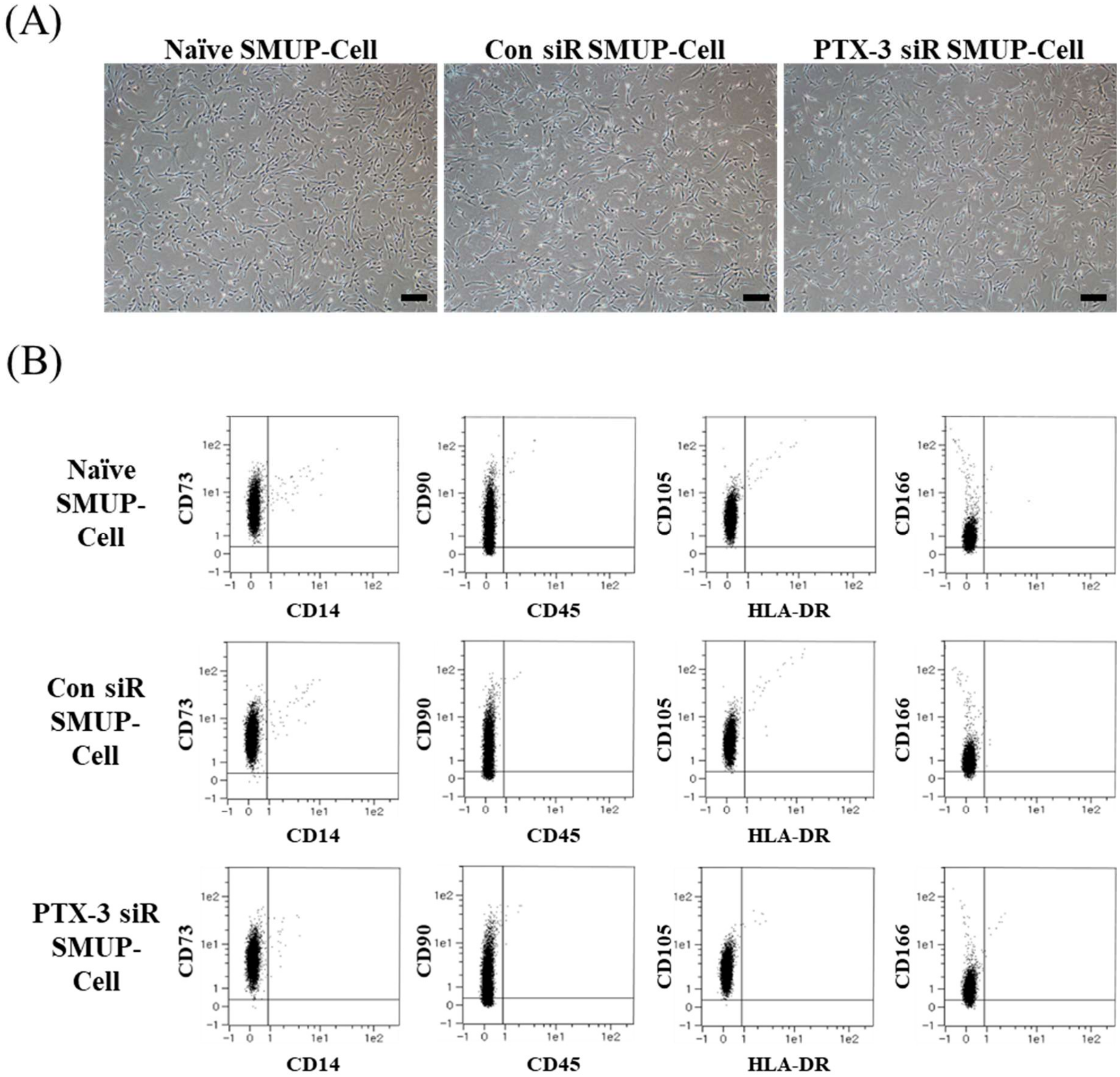

Figure S3. **Comparison of MSC characteristics based on PTX-3 expression. SMUP-Cell under three conditions were prepared.** (A) Morphology was analyzed in naïve, Con siR, and PTX-3 siR by microscope. All SMUP-Cell were observed as fibroblast like shape. (B) Surface antigen expression were tested by flow cytometry. Three condition cells were strongly positive for MSC specific marker CD73, CD90, CD105, and CD166, and they were negative for CD14, CD45, and HLA-DR.

(A)

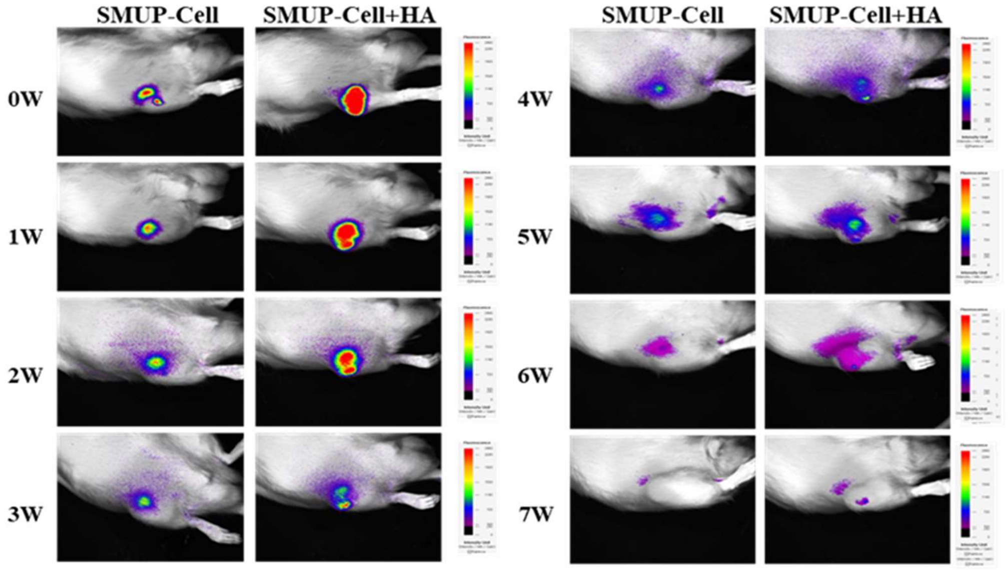

(B)

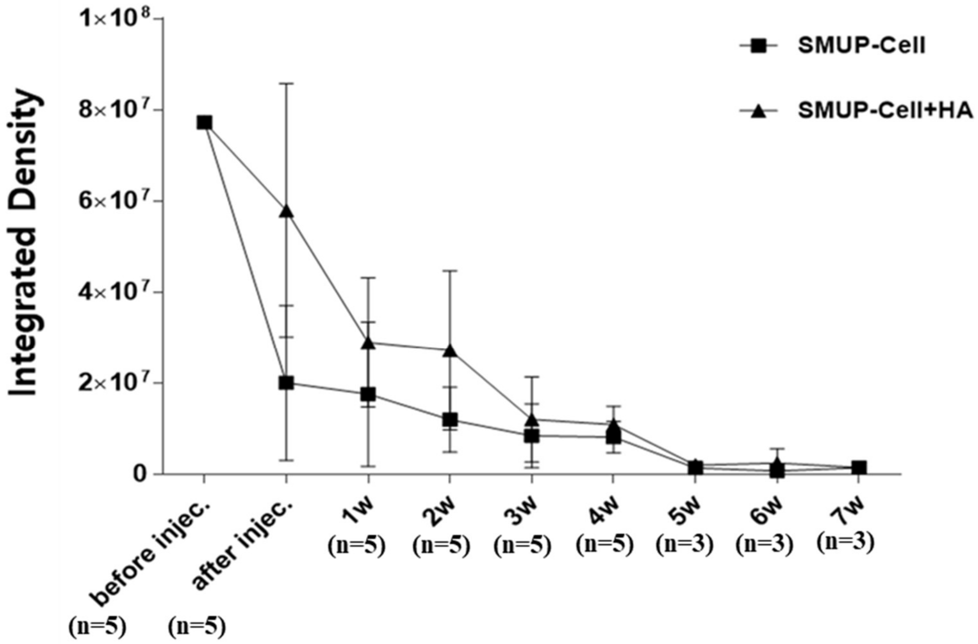

Figure S4. *In vivo* imaging of SMUP-Cells injected with or without HA into the MIA-induced OA rat model. (A) *In vivo* imaging. SMUP-Cells labeled with Neostatin 749 dye were transplanted into the OA rat knee and observed using a fluorescence imaging system for 7 weeks. Images were obtained at weeks 0 through week 7 after SMUP-Cell injection. (B) Integrated density was analyzed using the NEO imaging program (http://www.neoimaging.cn/). (A, B) Error bars represent the mean ± SEM. HA, Hyaluronic Acid; MIA, Monosodium Iodoacetate; Raw 264.7 cells; L, LPS-stimulated cells; SMUP, SMUP-Cell. Number (n) is per group.

Table S1. Small cell check

SMUP-Cell	Smaller cell portion (%, ≤10um)	
	Before filter	After filter
#1	35	91
#2	42	95
#3	50	93
#4	30	92
#5	35	90
mean±SD	38.4±7.7	93±1.9

Table S2. Bioreactor conditions

Condition	Initial seeding (cells/cm^2)	pH	Temperature (° C)	DO (%)	Culture time (day)
Range	500 ~ 3,000	7.2 ~ 7.4	37.0 ± 1.0	2 ~ 5	6 ~ 8

DO: dissolved Oxygen

Table S3. Basic information of SMUP-Cell characterization

SMUP-Cell	Surface marker		Differentiation
	Positive	Negative	
#1	Pass	Pass	Pass
#2	Pass	Pass	Pass
#3	Pass	Pass	Pass
#4	Pass	Pass	Pass
#5	Pass	Pass	Pass

UCBs were isolated from five independent donors (UCB #1 to 5). SMUP-Cell characterization was performed by assessing MSC surface marker expression and MSC differentiation capacity (positive: CD29, CD73, CD90, CD105, and CD166 ≥ 85%; negative: CD14, CD45, and HLA-DR ≤ 1.0%; differentiation: osteogenic, chondrogenic, adipogenic).

Table S4. Multi-differentiation condition

Differentiaiton		Reagent	
Osteogenesis	Induction	StemPro Osteogenesis Differentiation Kit	Gibco
	Staining	Alkaline Phosphatase Staining Kit II	Stemgent
		Von Kossa Method for Calcium Kit	Polysciences
Chondrogenesis	Induction	Dulbecco's modified Eagle's medium	Gibco
		TGFβ-3 (10ng/mL)	R&D systems
		BMP-6 (500ng/mL)	R&D systems
		Ascorbic Acid (50ug/mL)	Sigma-Aldrich
		ITS (50mg/mL, 1:100)	Sigma-Aldrich
		L-proline (40ug/mL)	Sigma-Aldrich
		Pyruvic acid (100ug/mL)	Sigma-Aldrich
		Dexametasone (100nM)	Sigma-Aldrich
	Staining	Safranin O	Sigma-Aldrich
		Collagen II	abcam
Adipogenesis	Induction	Dulbecco's modified Eagle's medium	Gibco
		FBS (10%)	Gibco
		3-isobutyl-1-methylxanthine (0.5mM)	Sigma-Aldrich
		Dexametasone (1mM)	Sigma-Aldrich
		Indomethacin (0.2mM)	Sigma-Aldrich
		h-Insulin (10mM)	Sigma-Aldrich
	Staining	Oil red O	Sigma-Aldrich
		BODIPY 493/503	Molecular Probes

Table S5. For proteome profile human array panel (55 spot)

Coordinate	Target	Coordinate	Target
A1, A2	Refence Spots	C17, C18	IL-8
A5, A6	Activin A	C19, C20	LAP(TGF-β1)
A7, A8	ADAMTS-1	C21, C22	Leptin
A9, A10	Angiogenin	C23, C24	MCP-1
A11, A12	Angiopoietin-1	D1, D2	MIP-1α
A13, A14	Angiopoietin-2	D3, D4	MMP-8
A15, A16	Angiostatin/Plasminogen	D5, D6	MMP-9
A17, A18	Amphiregulin	D7, D8	NRG1-β1
A19, A20	Artemin	D9, D10	Pentraxin 3 (PTX3)
A23, A24	Reference Spots	D11, D12	PD-ECGF
B1, B2	Coagulation Factor III	D13, D14	PDGF-AA
B3, B4	CXCL16	D15, D16	PDFG-AB/PDFG-BB
B5, B6	DPPIV	D17, D18	Persephin
B7, B8	EGF	D19, D20	Platelet Factor 4 (PF4)
B9, B10	EG-VEGF	D21, D22	PIGF
B11, B12	Endoglin	D23, D24	Prolactin
B13, B14	Endostatin/Collagen XVIII	E1, E2	Serpin B5
B15, B16	Endothelin-1	E3, E4	Serpin E1
B17, B18	FGF acidic	E5, E6	Serpin F1
B19, B20	FGF basic	E7, E8	TIMP-1
B21, B22	FGF-4	E9, E10	TIMP-4
B23, B24	FGF-7	E11, E12	Thrombospondin-1
C1, C2	GDNF	E13, E14	Thrombospondin-2
C3, C4	GM-CSF	E15, E16	uPA
C5, C6	HB-EGF	E17, E18	Vasohibin
C7, C8	HGF	E19, E20	VEGF
C9, C10	IGFBP-1	E21, E22	VEGF-C
C11, C12	IGFBP-2	F1, F2	Reference Spots
C13, C14	IGFBP-3	F23, F24	Negative Control
C15, C16	IL-1β		

Table S6. qRT-PCR primers and siRNA sequences

Gene		Primer sequence (5'→3')
Mouse GAPDH	Left	AGCTTGTCATCAACGGGAAG
	Right	TTTGATGTTAGTGGGGTCTCG
Mouse IL-6	Left	GCTACCAAACTGGATATAATCAGGA
	Right	CCAGGTAGCTATGGTACTCCAGAA
Mouse TNF-a	Left	TCTTCTCATTCCTGCTTGTGG
	Right	GGTCTGGGCCATAGAACTGA
Mouse IL-1β	Left	AGTTGACGGACCCCAAAAG
	Right	AGCTGGATGCTCTCATCAGG
Mouse ARG-1	Left	GAATCTGCATGGGCAACC
	Right	GAATCCTGGTACATCTGGGAAC
Human PTX-3	Left	GCGGTGCTAGAGGAGCTG
	Right	GGAATAAAATAGCTGTTTCACAACCT
Scramble siRNA	#1	UGGUUUACAUGUCGACUAA
	#2	UGGUUUACAUGUUGUGUGA
	#3	UGGUUUACAUGUUUUCUGA
	#4	UGGUUUACAUGUUUUCCUA
human PTX-3 siRNA	#1	GUGAAUUUGGACAACGAAA
	#2	CUGCAGUGUUGGCCGAGAA
	#3	GGUCAGGAGCACUCGGAAU
	#4	GGAUAGUGUUCUUAGCAAU

Table S7. Experimental design in vivo

Group (number)	MIA (2 mg/50 ul)	Cells/Sol1 (2.5×10^5/25ul)	HA (1%, 25ul)
Normal (n=6)	-	-	-
MIA (n=6)	+	-	-
MIA+HA (n=6)	+	-	+
MIA+HA+Naïve SMUP (n=6)	+	+	+
MIA+HA+Con siR SMUP (n=6)	+	+	+
MIA+HA+PTX-3 siR SMUP (n=6)	+	+	+

Table S8. Percent of weight on ipsilateral limb

Days	Normal	MIA	MIA+HA	MIA+HA		
				Naïve SMUP	Con siR SMUP	PTX-3 siR SMUP
**0**	49.97±0.39	50.03±0.18	50.32±0.40	50.99±0.43	49.97±0.52	49.74±0.41
**1**	50.12±0.32	33.72±1.20***	30.14±1.41***	30.84±1.67***	32.18±1.61***	32.4±1.49***
**4**	49.87±0.21	31.61±1.20***	28.99±2.21***	30.45±1.78***	31.62±2.33***	32.00±1.75***
**7**	49.80±0.30	30.39±1.67***	28.31±2.45***	37.30±2.26***#	31.16±2.29***	32.07±0.41***
**14**	50.64±0.19	27.24±2.14***	27.67±1.22***	45.89±0.44###	41.39±3.02***###	31.99±1.88***
**21**	49.98±0.17	27.17±1.24***	25.22±1.59***	48.23±1.26###	45.54±2.22*###	28.88±1.47***
**28**	49.32±0.45	25.31±1.01***	24.5±1.60***	48.4±1.08###	41.68±6.13###	28.39±1.27***

Data represent the mean ± SEM (n = 6 per group, Fig 6a result). *, $p < 0.05$, **, $p < 0.01$, ***, $p < 0.001$ (vs. normal), #, $p<0.05$, ##, $p<0.01$, ###, $p < 0.001$ (vs. MIA)
